# Autogenous Dermal-Fat Graft Reconstruction in Temporomandibular Joint Ankylosis: Functional Outcomes, Pain Reduction, and Scar Satisfaction

**DOI:** 10.3390/jcm15082924

**Published:** 2026-04-12

**Authors:** Özlem Gerginok Kaya, Ayça Özsoy, Sevil Altundağ Kahraman

**Affiliations:** Oral and Maxillofacial Surgery, Faculty of Dentistry, Gazi University, 06490 Ankara, Türkiye; ozlemgerginok@gmail.com (Ö.G.K.); skahraman99@yahoo.com (S.A.K.)

**Keywords:** TMJ disease, rehabilitation, pain assessment, donor site, scar quality

## Abstract

**Background**: This study aims to evaluate the functional improvement, pain reduction, and esthetic outcomes including scar satisfaction and donor-site morbidity following autogenous dermal-fat graft reconstruction for temporomandibular joint (TMJ) ankylosis. **Methods**: This retrospective clinical study included 12 adults with TMJ ankylosis treated surgically with autogenous dermal fat graft reconstruction. Outcomes measured included pre- and postoperative maximum mouth opening (MMO), pain intensity via the Visual Analog Scale (VAS) at postoperative day 2, week 1, and month 6, and scar-satisfaction scores. Changes in functional and esthetic outcomes over time and the correlation between preoperative mouth opening and postoperative pain were analyzed using appropriate parametric or non parametric tests and correlation analyses. **Results**: Mean MMO significantly improved from 8.17 ± 6.72 mm to 29.58 ± 5.68 mm (*p* = 0.002). Mean VAS pain scores declined steadily from 5.50 ± 1.88 at day 2 to 1.50 ± 1.73 at 6 months (*p* < 0.001). A strong negative correlation was found between preoperative MMO and postoperative pain at all intervals (*p* < 0.001), indicating that more severe preoperative restriction is associated with higher postoperative pain. Esthetic satisfaction was high (patients: 2.08 ± 1.38; surgeons: 1.58 ± 1.00), and donor-site morbidity was minimal. No re-ankylosis occurred during the 36-month mean follow-up. **Conclusions**: Autogenous dermal fat grafting for TMJ ankylosis provided favorable functional recovery, esthetic outcomes, and manageable donor site morbidity. Analysis suggests that restricted preoperative mouth opening is associated with greater postoperative pain, supporting perioperative analgesia and physiotherapy for patients.

## 1. Introduction

Temporomandibular joint (TMJ) ankylosis is a pathological condition characterized by fusion of the mandible to the glenoid fossa by fibrous or bony tissue. This condition impairs mastication, speech, oral hygiene, and daily activities, and may become potentially life-threatening in situations where emergency airway management is required [1]. Moreover, in growing patients, ankylosis can result in facial asymmetry and dentofacial deformities [2]. Various etiological factors have been reported, with trauma being the most common, followed by infections, arthritis, previous surgical interventions, congenital deformities, and idiopathic conditions [3,4,5]. The epidemiological data reveals significant geographic variation in TMJ ankylosis occurrence. The condition shows notably high prevalence in regions of Asia and Africa [6], while incidence has been declining in Europe and North America but remains common in developing countries [4,7]. In India, the incidence reaches 0.46 per 1000 cases in the 3–15 age group, with 128 cases identified among 1607 pediatric patients [6]. A Nigerian study reported TMJ ankylosis in 1.6% of 3596 mandibular condylar fractures over 16 years [8]. The diagnosis is established through clinical examination and imaging techniques such as CT or MRI [2]. The primary goals of TMJ ankylosis management are to restore adequate mouth opening, prevent re-ankylosis, correct asymmetry, and reduce pain [1].

Various surgical approaches have been described, including gap arthroplasty, interpositional arthroplasty, and total joint reconstruction, with interpositional materials such as temporalis muscle flap, costochondral grafts, dermal fat grafts, skin grafts, and alloplastic implants [9]. Although patient-specific total joint reconstruction has become a preferred treatment for adult TMJ ankylosis due to its superior long-term functional stability and lower recurrence rates, autogenous dermal-fat grafting remains a relevant biological alternative. It is particularly valuable in cases where cost-effectiveness, patient preference for autogenous tissue, or lack of access to custom-made prostheses are primary considerations. Dermal fat grafts are preferred due to their availability, low donor site morbidity, low immunogenicity, vascular structure, and anti-fibrotic properties, and the presence of stem cells in adipose tissue that can support healing [10]. In the present study, dermal-fat grafting was selected in cases where facial symmetry could be achieved following removal of the ankylosed mass, as the limitation was primarily due to joint immobility and not associated with significant ankylosis-related mandibular growth disturbance, which would otherwise necessitate reconstruction with a patient-specific total joint reconstruction, and where cost considerations also favored the use of autogenous tissue.

While the use of dermis-fat grafts in TMJ ankylosis surgery remains, the available literature provides only limited information on patient-reported outcomes and pain. The present study aims to evaluate the association between restricted preoperative mouth opening and postoperative pain intensity, which has not been quantitatively or systematically assessed to date, as well as secondary outcomes including patient satisfaction, scar characteristics, and pain at both the recipient and donor sites.

## 2. Patients and Methods

### 2.1. Patient Data

This single-center retrospective cohort study was approved by the Institutional Review Board (IRB, No. E.5375 and date of approval 13 February 2020) of this hospital and adhered to the principles of the Declaration of Helsinki. Due to the retrospective nature of the study and the use of de-identified data, the requirement for informed consent was waived by the IRB. Written informed consent for surgery and the use of anonymized data and images for research and publication purposes was obtained from all patients. This retrospective study included 12 patients who underwent autogenous dermal-fat grafting for the surgical management of TMJ ankylosis at the Department of Oral and Maxillofacial Surgery, Gazi University Faculty of Dentistry, between 2015 and 2019. All procedures were performed by the same surgical team.

Data obtained from patient records and follow-up examinations included sociodemographic characteristics, etiology, ankylosis classification, postoperative pain, scar length, mouth opening, and nerve sensitivity. The diagnosis was retrospectively verified based on clinical notes and radiographic findings observed on pre-existing cone-beam computed tomography (CBCT) images. Ankylosis was classified as osseous, fibrous, or fibro-osseous and further categorized according to the Topazian and Sawhney classification systems.

### 2.2. Sample Size

An a priori sample size analysis was performed using G*Power 3.1.9.7. Considering the rarity of TMJ ankylosis and the limited sample sizes reported in previous studies, effect size values were determined based on findings from the literature rather than arbitrary assumptions. Previous studies have demonstrated substantial postoperative improvements in mouth opening following surgical treatment of TMJ ankylosis. For example, Karamese et al. reported an increase in maximum mouth opening (MMO) from approximately 5.6 mm preoperatively to 29.5 mm postoperatively, while Dimitroulis reported an increase from 15.6 mm to 35.7 mm. These findings indicate marked clinical improvements following surgical intervention [11,12]. Based on these substantial improvements, large effect sizes were considered appropriate for the present analysis. For the repeated assessment of Visual Analog Scale (VAS) scores across three time points, a repeated-measures ANOVA model was used. Assuming a large effect size (f=0.40), a type I error rate of α=0.05, and a statistical power of 80%, the minimum required sample size was calculated as 12 participants. Additionally, for the comparison of preoperative and postoperative measurements within the same individuals, a paired-samples design was considered. Based on the magnitude of improvement reported in previous studies, a large effect size ($d_{z}=0.80$) was used (α=0.05, power = 0.80), yielding a required sample size of 12 participants. Since both analytical approaches resulted in the same minimum sample size requirement, the final sample size was determined as at least 12 participants.

### 2.3. Inclusion and Exclusion Criteria

Inclusion criteria were: (1) Age ≥18 years; (2) diagnosis of TMJ ankylosis requiring surgical treatment; (3) reconstruction with autogenous dermal-fat graft; and (4) availability of complete clinical and follow-up records. Two patients who had previously undergone ankylosis surgery at another center and presented with re-ankylosis were also included. A detailed history was obtained from all patients to determine etiological factors and the cases were classified accordingly.

Patients who did not meet any of the above inclusion criteria were excluded.

### 2.4. Surgical Technique

Preoperative MMO was measured in all patients during clinical and radiographic examination. Surgical treatment was performed under general anesthesia. Fiberoptic nasal intubation, considered the gold standard for difficult airway management in ankylosis patients [13], was used in 11 cases, while 1 patient underwent tracheostomy due to a history of previous tracheostomy.

After routine skin preparation and local anesthetic infiltration, a preauricular incision was made to expose the ankylosed joint. The ankylosed mass and degenerated disc were excised. Intraoperative mouth opening was measured and a bilateral coronoidectomy was performed when MMO remained <35 mm.

**Figure 1:**
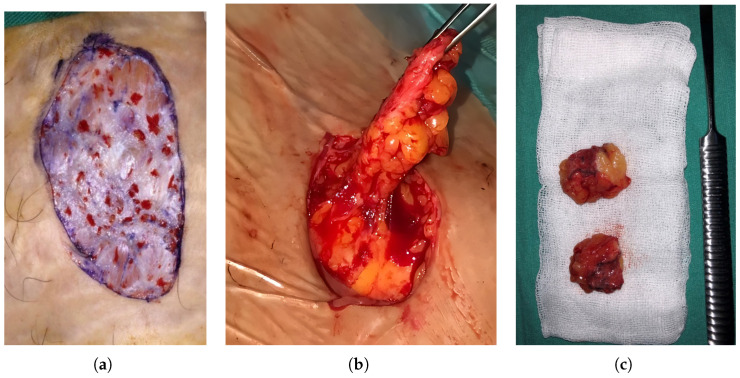
(**a**) The epidermis layer was carefully removed from the fat graft. (**b**) Harvesting of the dermal fat graft from the abdominal region. (**c**) Dermal fat grafts from the abdominal region.

**Figure 2:**
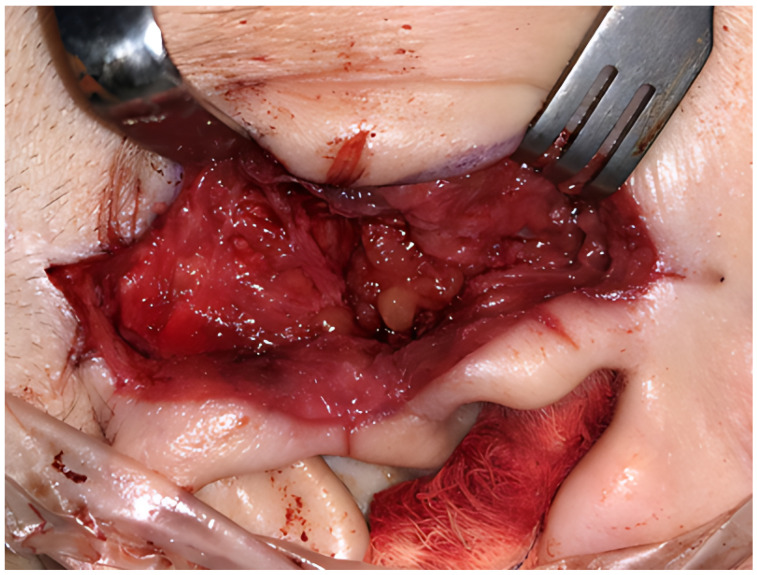
Placement of dermal-fat grafts in the created joint space.

Dermal-fat grafts were simultaneously harvested from the abdominal region through an elliptical incision. The epidermis was carefully removed (Figure 1 and Figure 2) and grafts measuring approximately 4 × 2 cm were prepared (Figure 1).

The grafts were then placed in the surgically created joint spaces (Figure 2). Donor and recipient sites were closed in layers. A pressure dressing was applied over the donor site for 3–4 days to prevent hematoma or seroma formation. All patients received standard postoperative antibiotic and analgesic therapy. The sutures were removed on postoperative days 10–14.

Passive mouth-opening exercises were initiated on postoperative day 3, after reduction of edema, and followed by progressive physiotherapy over the subsequent days. The patients were examined at the 1 week, 3 months, and 6 months postoperatively.

### 2.5. Outcome Measures and Data Collection

The following parameters were recorded:

- Pain intensity, assessed with the VAS on postoperative day 2, week 1, and month 6.
- Duration of mouth-opening exercises and physiotherapy.
- Scar characteristics (length, width, appearance, texture, keloid/hypertrophic/normal) at both TMJ and donor sites.
- Patient satisfaction with scar appearance (patient and surgeon scores).
- Patient satisfaction regarding treatment outcome and recommendation to others, assessed using a structured binary questionnaire (yes/no responses) during follow-up visits.
- Pre- and postoperative MMO.
- Complications, including facial nerve paralysis, infection, hematoma, and wound dehiscence.

Demographic data (age, gender, date of operation, type and classification of ankylosis) and follow-up findings were obtained from detailed postoperative records and clinical examinations. Patients were evaluated at predefined postoperative intervals (day 2, week 1, month 3, and month 6), during which VAS pain scores, and scar characteristics were systematically recorded. Beyond 6 months (mean follow-up: 36 months, range 12–60 months), follow-up visits primarily involved clinical assessment of complications, functional status, and signs of re-ankylosis, with quantitative measurements repeated only when clinically indicated.

### 2.6. Statistical Analysis

Statistical analysis was performed using SPSS version 20.0, and a *p*-value < 0.05 was considered statistically significant. Data were presented as mean ± standard deviation, median (min–max), and number (percentage), as appropriate. The normality of data distribution was assessed using the Shapiro–Wilk test, which is considered more reliable for small sample sizes (n<50). Due to the limited sample size and non-normal distribution of the data, non-parametric methods were preferred. For comparisons of preoperative and postoperative repeated measurements, the Wilcoxon Signed Ranks test was used. For repeated measurements across three time points, the Friedman two-way analysis was applied, and Bonferroni-adjusted post hoc comparisons were performed when significant differences were detected. The relationships between preoperative mouth opening and postoperative VAS scores were evaluated using Spearman correlation analysis. In accordance with current recommendations for statistical reporting in clinical and dental research [14], effect sizes were reported alongside *p*-values and interpreted based on Cohen’s criteria. For the Wilcoxon Signed Ranks test, the effect size (*r*) was calculated as $r=Z/\sqrt{N}$. Kendall’s *W* was used as the effect size measure for the Friedman test, and Spearman’s rho (ρ) was reported for correlation analyses. To enhance the interpretability of the results, 95% confidence intervals (CIs) for effect sizes and correlation coefficients were estimated using bootstrap resampling (5000 iterations). Although the primary analyses were conducted in SPSS, CI estimation was performed in R (Version 4.4.1, R Foundation for Statistical Computing, Vienna, Austria), since SPSS does not provide built-in procedures for calculating confidence intervals for non-parametric effect size measures (e.g., *r* for the Wilcoxon signed-rank test and Kendall’s *W*). Therefore, R was used as a supplementary tool to obtain robust bootstrap-based confidence intervals. Confidence intervals were successfully obtained for the Friedman test and Spearman correlation analyses. However, for the Wilcoxon signed-rank test, in cases where all non-zero paired differences were in the same direction, bootstrap resampling yielded identical effect size estimates across all iterations, resulting in zero variance in the bootstrap distribution. Consequently, confidence intervals could not be computed for the Wilcoxon effect size in these comparisons.

## 3. Results

### 3.1. Patient Characteristics

A total of 12 patients (9 females, 3 males) were included, comprising 10 unilateral and 2 bilateral ankylosis cases. The mean age was 35 years (range 18–55) and the mean follow-up period was 36 months (range 12–60). The most common etiology was trauma (7 patients), followed by head and neck radiotherapy (3 patients), childhood ear infection (1 patient) and rheumatoid arthritis (1 patient). According to the Topazian classification, 64.3% of ankylosed joints were stage 1, 21.4% stage 2, and 14.3% stage 3. According to the Sawhney classification, 35.7% were type 1, 28.6% type 2, 28.6% type 3, and 7.1% type 4.

### 3.2. Surgical Details and Hospital Course

Unilateral surgery was performed in 83.3% of the cases and bilateral surgery in 16.7%. Bilateral coronoidectomy was required in 33.3% of the patients. Condylar reshaping was performed in 25% of the patients. The mean hospital stay was 1.42 ± 0.68 days. All sutures were removed within 10–14 days and no wound dehiscence was observed. The mean duration of mouth-opening exercises and physiotherapy was 2.83 ± 1.27 months. By the 6-month follow-up evaluation, quantitative pain scores had significantly subsided, with the majority of patients reporting minimal to no residual discomfort. At later follow-up visits, no patient reported clinically relevant persistent pain on routine clinical questioning.

### 3.3. Scar Characteristics and Patient Satisfaction

Scar characteristics for both the TMJ region and dermal-fat graft donor site are summarized in (Table 1), presenting mean ± standard deviation values along with minimum–maximum ranges for scar length, width, and appearance scores. In the TMJ region, the mean scar length was measured as 50.83 ± 7.93 mm (40–70). For the dermal-fat graft donor site, the mean scar length was 60.83 ± 18.32 mm (40–90) and the mean scar width was 0.36 ± 0.20 mm (0.20–0.80). Regarding scar appearance scores, patient assessments yielded a mean score of 2.08 ± 1.38 (0–4), while surgeon assessments demonstrated a mean score of 1.58 ± 1.00 (0–3). The distribution of scar color, scar appearance, and scar consistency is presented as frequencies (n) and percentages (%). In the TMJ region, all scars exhibited tissue-matched color (100%), normal appearance (100%), and soft consistency (100%). In the dermal-fat graft donor site, scar color distribution was as follows: tissue-matched color in 75% (n = 9), light color in 8.3% (n = 1), and dark color in 16.7% (n = 2) of cases. Notably, both regions demonstrated normal scar appearance (100%) and soft scar consistency (100%). In general, the scar characteristics were generally acceptable and contributed to high patient satisfaction. All patients reported satisfaction with the surgical outcome and stated that they would recommend the procedure to others.

### 3.4. Mouth Opening Outcomes

The difference between preoperative and postoperative MMO values was analyzed using the Wilcoxon Signed Ranks test (Table 2). The mean preoperative MMO was 8.17 ± 6.72 mm (median: 9, range: 1–20), which significantly increased to 29.58 ± 5.68 mm (median: 31, range: 20–37) in the postoperative period. This increase was found to be statistically significant (Z=−3.062, p=0.002). These findings demonstrate that all patients showed a substantial increase in mouth opening capacity following treatment. The effect size was large (r=0.88), according to Cohen’s criteria [14], indicating a strong and clinically meaningful improvement in mouth opening following treatment. For the Wilcoxon Signed Ranks test, the effect size (*r*) was calculated by dividing the Z value by the square root of the sample size ($r=Z/\sqrt{N}$), as recommended in the literature.

### 3.5. Pain Outcomes

Postoperative pain was assessed using the VAS pain scores at three time points. According to the results, the mean VAS score on postoperative day 2 (T1) was 5.5 ± 1.88 (median: 5, range: 3–9), which decreased to 3.25 ± 1.36 (median: 3, range: 1–5) at week 1 (T2), and further declined to 1.5 ± 1.73 (median: 1, range: 0–6) at month 6 (T3) (Table 3). The change in pain scores over time was evaluated using the Friedman two-way analysis, and a statistically significant difference was found between the repeated measurements ($\chi^{2}=22.167$, p<0.001). Post hoc Bonferroni analysis revealed that the VAS scores at month 6 (T3) were significantly lower than those at day 2 (T1) (p<0.001), and the scores at week 1 (T2) were also significantly lower than those at day 2 (T1) (p=0.024). No significant difference was observed between week 1 (T2) and month 6 (T3). The effect size was large (Kendall’s W=0.924), according to Cohen’s criteria, indicating a strong reduction in pain over time. The 95% confidence interval ranged from 0.812 to 1.000.

### 3.6. Correlation Between Preoperative Mouth Opening and Postoperative Pain

The relationship between preoperative MMO and pain (VAS) was evaluated using Spearman correlation analysis. The results demonstrated a statistically significant and strong negative correlation between preoperative MMO and VAS scores at all postoperative time points. Specifically, strong negative correlation was observed on day 2 (ρ=−0.930, p<0.001, 95% CI: −0.9855 to −0.7705), week 1 (ρ=−0.952, p<0.001, 95% CI: −0.9980 to −0.8111), and month 6 (ρ=−0.891, p<0.001, 95% CI: −0.9708 to −0.6969). According to Cohen’s criteria, all correlations were in the strong range. These findings indicate that as mouth opening increases, postoperative pain levels decrease significantly across all follow-up periods (Table 4).

### 3.7. Long-Term Surveillance and Recurrence

During extended follow-up (mean 36 months, range 12–60), no cases of re-ankylosis were observed. All patients maintained functional mouth opening with sustained satisfaction. Clinical assessment confirmed preserved functional capacity without progressive restriction or major complications.

## 4. Discussion

TMJ ankylosis represents a significant functional disability that requires surgical intervention to restore jaw function and quality of life. While various interpositional materials have been proposed to prevent re-ankylosis following gap arthroplasty, the optimal choice remains debatable. This study evaluated the clinical outcomes of autogenous dermal-fat grafting in the surgical management of TMJ ankylosis in 12 patients over a mean follow-up period of 36 months.

According to previous reports, trauma was the most common etiology of TMJ ankylosis in this series, followed by radiotherapy, childhood ear infection, and rheumatoid arthritis [3,7,15,16,17,18,19]. The predominance of female patients also aligns with the distribution reported in the literature [20,21] Osseous ankylosis was the most common type, which is also in agreement with earlier clinical series [20].

The present study achieved a mean postoperative MMO of 29.58 ± 5.68 mm at 6 months of follow-up, representing a clinically significant improvement from the preoperative baseline of 8.17 ± 6.72 mm. Importantly, this gain reflects not only a statistical difference but also a functional milestone enabling the restoration of daily activities such as effective mastication and speech, both fundamental to a patient’s quality of life. This outcome is consistent with Rahman et al., who reported a mean MMO of 30.1 mm at 6 months using abdominal dermal fat grafts in patients with comparable preoperative severity (mean preoperative MMO: 5.1 mm) [15]. Although Kilinskaite et al.’s systematic review reported higher MMO values (40.0 ± 2.7 mm at 3 months, 40.80 ± 4.26 mm at 6 months, 41.9 ± 4.0 mm at 12 months, and 43.5 mm beyond 12 months) [22], these findings derive from heterogeneous populations. The variability in reported MMO outcomes across studies is therefore more likely attributable to differences in case complexity rather than graft efficacy. Notably, our cohort included two patients with re-ankylosis following previous failed surgery and 35.7% of patients with Sawhney Type 1 ankylosis representing a more severe clinical subset. Despite these challenging baseline characteristics, all patients achieved substantial functional improvement with no recurrence at final follow-up, further supporting the effectiveness of abdominal dermal fat grafts even in complex cases. However, these findings should be interpreted with caution given the limited sample size, which may influence the precision of the estimated treatment effect.

Patient-specific total joint reconstruction has demonstrated long-term functional stability and low recurrence rates in adult TMJ ankylosis, with custom-made prostheses offering precise fit and accuracy, particularly in cases of severe anatomical disruption that demand patient-specific solutions [4,23]. Despite these advantages, autogenous dermal-fat grafting remains a compelling biological alternative. It is particularly valuable in cases where cost-effectiveness, patient preference for autogenous tissue, or lack of access to custom-made prostheses are primary considerations.

Gap arthroplasty remains a widely accepted technique when complete removal of the ankylotic mass and an adequate gap (≥1 cm) are achieved, with complete resection—first emphasized by Humphrey—remaining the cornerstone of treatment [24,25]. In the present series, gap arthroplasty combined with abdominal dermal fat interposition resulted in zero reankylosis during follow-up, supporting the validity of our surgical protocol and addressing a key limitation of plain gap arthroplasty, which has reported recurrence rates of 13.9% despite adequate gap creation [5]. Our choice of dermis fat interpositional arthroplasty rather than gap arthroplasty alone was based on two critical factors: (1) the established superiority of interpositional materials in preventing heterotopic bone formation and (2) the specific advantages of dermal fat over alternative materials [5,22].

The absence of reankylosis in our cohort is consistent with previous reports, as Wolford documented no recurrence in fat-grafted patients with reoperation required in non-grafted cases [26], and Andrade et al. similarly demonstrated zero recurrence over a 3-year follow-up in a prospective randomized trial [27]. This consistency across studies supports the concept that interpositional materials may modify the biological environment of the resection gap by limiting pluripotential mesenchymal cell migration and subsequent heterotopic ossification [10]. Nevertheless, the absence of a control group in the present study limits direct attribution of this outcome specifically to dermal-fat grafting.

The abdomen is the most common donor site for autogenous fat harvesting, typically via suprapubic or periumbilical incisions, with the possibility of using pre-existing scars (e.g., cesarean, hysterectomy, appendectomy). Other potential donor sites include the hips, thighs, buccal fat pad, and breasts [22,28]. Beyond recurrence prevention, dermal fat from the lower abdomen or iliac crest provides superior functional outcomes compared to buccal fat pad or temporalis fascia, with Kilinskaite et al.’s systematic review of 369 patients demonstrating the greatest maximal mouth opening improvements, lowest pain intensity, and least volumetric contraction [22]. The technique most widely recommended involves removing the epidermis and transplanting the graft with the dermal layer intact to stabilize the fat, reduce fragmentation, and facilitate adaptation [12,15]. In this study, all grafts were harvested from the abdominal region and transplanted with the dermal layer preserved, resulting in acceptable scar characteristics and high patient satisfaction.

The VAS pain scores in the present study decreased from a mean of 5.5 ± 1.88 on postoperative day 2 to 3.25 ± 1.36 at week 1 and 1.5 ± 1.73 at 6 months, demonstrating a marked and sustained reduction in pain intensity over time. This progressive reduction in pain intensity is consistent with findings from comparable investigations using dermal fat grafts [15,16,22].

Beyond its immediate clinical relevance, postoperative pain represents a critical determinant of functional recovery. The recurrence of TMJ ankylosis is largely driven by two key factors: the enhanced propensity for heterotopic bone formation following surgery and the inhibition of effective physiotherapy due to postoperative pain [15]. In this regard, Ezoe et al. underscored the critical role of sustained physiotherapy in minimizing relapse over a 24-month follow-up period [29], while systematic reviews further indicate that postoperative physiotherapy significantly improves maximal mouth opening and alleviates pain following open TMJ procedures [30].

In line with these considerations, the present study demonstrated a significant negative correlation between preoperative maximal mouth opening and postoperative pain intensity at all measured time points (day 2, week 1, and month 6), with patients presenting with more severe preoperative restriction experiencing higher postoperative pain levels. Several mechanisms may explain this observation. First, chronic TMJ ankylosis leads to adaptive shortening of masticatory muscles and periarticular soft tissues, requiring more extensive surgical release and resulting in greater tissue trauma. Second, prolonged immobilization may contribute to altered pain processing, potentially increasing postoperative pain perception. Third, more severe restriction is often associated with more extensive ankylosis, necessitating more aggressive surgical intervention [4,29].

Clinically, this finding suggests that patients with severe preoperative restriction may represent a higher-risk subgroup requiring optimized perioperative pain management strategies and more intensive, closely supervised physiotherapy programs to maximize rehabilitation adherence and functional outcomes [5,27]. However, this implication should be considered exploratory until validated in larger prospective cohorts.

In this context, long-term success after TMJ ankylosis surgery hinges not only on the selected surgical technique, but also on the timing of intervention, and well-structured postoperative rehabilitation emerges as a pivotal factor in the management. In this context, Olate.et.al. argue that customised TMJ replacement should be performed before the disease progresses to an end-stage, particularly when marked mandibular morphological changes are present, because early surgery can prevent irreversible skeletal deformities [31].

This study has several limitations. The small sample size and retrospective design may limit the generalizability of the findings. The structured quantitative follow-up was limited to the first 6 months, which precludes detailed assessment of long-term changes in functional outcomes. In addition, the absence of a control group prevents direct comparison across treatment modalities, and scar satisfaction was evaluated using subjective measures. Despite these limitations, the study provides clinically relevant preliminary data on functional, pain-related, and cosmetic outcomes following dermal-fat grafting in TMJ ankylosis. Further prospective studies with larger cohorts and standardized long-term quantitative follow-up are needed to validate these findings.

## 5. Conclusions

Autogenous dermal-fat grafting is a safe and effective technique for managing TMJ ankylosis, providing substantial improvements in mouth opening, low donor site morbidity, and high patient satisfaction with scar appearance. An exploratory association was observed between more restricted preoperative mouth opening and higher postoperative pain, suggesting that patients with severe preoperative restriction may benefit from enhanced perioperative analgesia and intensive physiotherapy. However, given the retrospective design, modest sample size, and multifactorial nature of postoperative pain, this finding requires cautious interpretation and validation in larger prospective cohorts before definitive clinical recommendations can be established. The present findings underscore the critical importance of tailored rehabilitation protocols in achieving optimal functional outcomes.

## Figures and Tables

**Table 1:** Distribution of scar characteristics and appearance scores of the TMJ area and dermal-fat graft donor site in terms of descriptive statistics.

Variable		Mean ± SD	Min–Max
Temporomandibular Joint Area—Scar Length (mm)		50.83 ± 7.93	40–70
Dermal Fat Graft Site—Scar Length (mm)		60.83 ± 18.32	40–90
Dermal Fat Graft Site—Scar Width (mm)		0.36 ± 0.20	0.20–0.80
Scar Appearance (0-Acceptable, 10-Ugly)—Patient		2.08 ± 1.38	0–4
Scar Appearance (0-Acceptable, 10-Ugly)—Surgeon		1.58 ± 1.00	0–3
		**n**	**%**
Temporomandibular Joint Area—Scar Color	Light Color	-	-
	Skin-Colored	12	100
	Dark Color	-	-
Dermal Fat Graft Site—Scar Color	Light Color	1	8.3
	Skin-Colored	9	75
	Dark Color	2	16.7
Temporomandibular Joint Area—Scar Appearance	Keloid	-	-
	Hypertrophic	-	-
	Normal	12	100
Dermal Fat Graft Area—Scar Appearance	Keloid	-	-
	Hypertrophic	-	-
	Normal	12	100
Temporomandibular Joint Area—Scar Texture	Soft	12	100
	Firm	-	-
Dermal Fat Graft Area—Scar Texture	Soft	12	100
	Firm	-	-

**Table 2:** Comparison of preoperative and postoperative maximum mouth opening.

Variable	Mean ± SD (mm)	M. (Min–Max) (mm)	Z	*p*
Preoperative	8.17 ± 6.72	9 (1–20)	−3.062	0.002 *
Postoperative	29.58 ± 5.68	31 (20–37)		
Difference	21.42 ± 7.27	10–33		

* *p* < 0.05, Z: Wilcoxon Signed Ranks Test Statistics, Effect size (r) = 0.88.

**Table 3:** Assessment of postoperative pain scores using the Visual Analogue Scale (VAS).

	Mean ± SD (mm)	M. (Min–Max) (mm)	Friedman Two-Way Analysis	*p*	Bonferroni
Day 2 (T1)	5.5 ± 1.88	5 (3–9)	22.167	<0.001 *	T3 < T1 (<0.001 *) T2 < T1 (0.024 *)
Week 1 (T2)	3.25 ± 1.36	3 (1–5)			
Month 6 (T3)	1.5 ± 1.73	1 (0–6)			

* *p* < 0.05, Effect size (Kendall’s W) = 0.924, indicating a very large effect, 95% CI = [0.812, 1].

**Table 4:** Correlation between preoperative maximum mouth opening and pain (VAS) at differet follow-up intervals.

VAS	Correlation Coefficient (*ρ*)	*p* Value
Day 2	−0.930 **	<0.001 *
Week 1	−0.952 **	<0.001 *
Month 6	−0.891 **	<0.001 *

* *p* < 0.05, ** *p* < 0.01, ρ: Spearman correlation coefficient.

## Data Availability

The data are available upon request from the corresponding author.
